# Carbon-Based Flexible and All-Solid-State Micro-supercapacitors Fabricated by Inkjet Printing with Enhanced Performance

**DOI:** 10.1007/s40820-016-0119-z

**Published:** 2016-12-08

**Authors:** Zhibin Pei, Haibo Hu, Guojin Liang, Changhui Ye

**Affiliations:** 1 Anhui Key Laboratory of Nanomaterials and Technology, Key Laboratory of Materials Physics, Institute of Solid State Physics, Chinese Academy of Sciences, Hefei, 230031 People’s Republic of China; 2 University of Science and Technology of China, Hefei, 230026 People’s Republic of China; 3 Hubei Collaborative Innovation Center for Advanced Organic Chemical Materials, Ministry-of-Education Key Laboratory for the Green Preparation and Application of Functional Materials, Faculty of Materials Science and Engineering, Hubei University, Wuhan, 430062 People’s Republic of China

**Keywords:** Inkjet printing, Flexible devices, Graphene oxide (GO), Carbon-based ink, Micro-supercapacitors

## Abstract

**Abstract:**

By means of inkjet printing technique, flexible and all-solid-state micro-supercapacitors (MSCs) were fabricated with carbon-based hybrid ink composed of graphene oxide (GO, 98.0 vol.%) ink and commercial pen ink (2.0 vol.%). A small amount of commercial pen ink was added to effectively reduce the agglomeration of the GO sheets during solvent evaporation and the following reduction processes in which the presence of graphite carbon nanoparticles served as nano-spacer to separate GO sheets. The printed device fabricated using the hybrid ink, combined with the binder-free microelectrodes and interdigital microelectrode configuration, exhibits nearly 780% enhancement in areal capacitance compared with that of pure GO ink. It also shows excellent flexibility and cycling stability with nearly 100% retention of the areal capacitance after 10,000 cycles. The all-solid-state device can be optionally connected in series or in parallel to meet the voltage and capacity requirements for a given application. This work demonstrates a promising future of the carbon-based hybrid ink for directly large-scale inkjet printing MSCs for disposable energy storage devices.

**Graphical Abstract:**

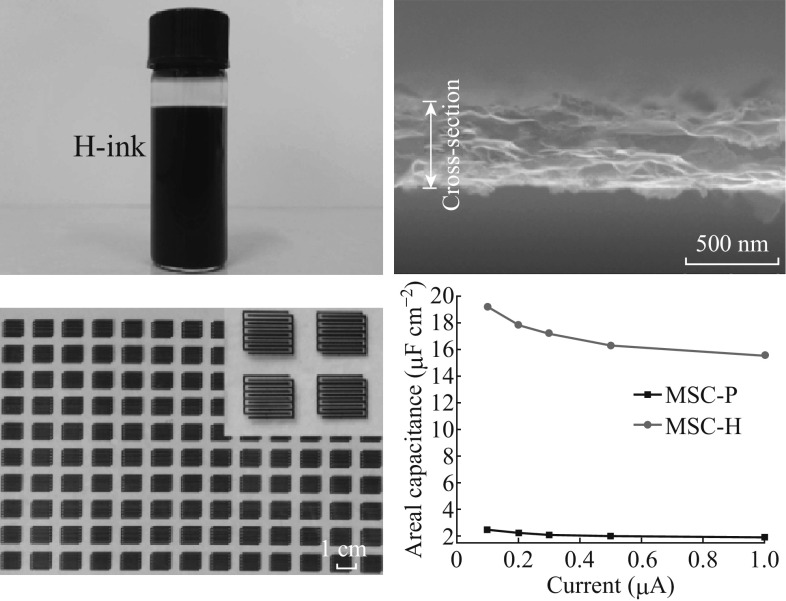

**Electronic supplementary material:**

The online version of this article (doi:10.1007/s40820-016-0119-z) contains supplementary material, which is available to authorized users.

## Highlights

- Flexible and all-solid-state micro-supercapacitors (MSCs) were fabricated by inkjet printing using carbon-based hybrid ink composed of graphene oxide (GO) and commercial pen ink.
- The as-obtained MSCs based on hybrid ink exhibit great enhancement in areal capacitance, flexibility and cycling stability compared with that of pure GO ink.
- This work provides a promising strategy for large-scale preparation of low-cost, lightweight, and flexible/wearable energy storage devices with carbon-based hybrid ink.

## Introduction

Significant advances in nanotechnology have greatly prompted the development of maintenance-free microelectronic devices such as wireless microsensors, implantable medical devices, nanorobotics, and active radio frequency identification tags, which have ultimately stimulated the rapid development of new concept and sufficiently compact energy storage systems [1–3]. In this respect, miniaturized supercapacitors (SCs), also called micro-SCs (MSCs), which can be fabricated directly on plastic or paper substrates and integrated with other microelectronic device, are attracting more and more attention as an important class of energy storage devices [4–11]. According to the charge storage mechanism as well as active materials used, SCs/MSCs generally have three types. The first is called electrochemical double-layer capacitors (EDLCs), in which the charge is stored by the surface charge separation at the electrode/electrolyte interface. The second is pseudo-capacitors or redox-capacitors, which is using fast and reversible surface faradic redox reactions for charge storage. The last are hybrid capacitors which consist of both EDLCs and pseudo-capacitors in a single device [4, 5]. Because neither of these two surface charge storages involve diffusion of ions within the inner bulk region of electrode active materials, SCs/MSCs possess a higher power density which is an order of magnitude larger (10,000 W kg^−1^) than that of regular batteries (e.g., lithium-ion batteries). In spite of the relatively lower energy density, SCs/MSCs can deliver an energy density in two orders of magnitude higher (10 Wh kg^−1^) than that of conventional capacitors. Furthermore, unlike batteries, due to their highly reversible charge storage process, SCs/MSCs have longer life-cycle that can often achieve up to millions of charge–discharge cycles without energy storage capacity loss. With these excellent performances, SCs/MSCs have been widely acknowledged as a promising compatible power source to complement or replace micro-batteries to provide the required power and energy for maintenance-free microelectronic devices [12–16].

Conventional MSCs usually follow the planar 2D architecture of thin film micro-batteries, consisting of thin-film electrodes that are stacked on top of each other with a separator in between [17–23]. Although the configuration with sandwich design is applicable to most of the electroactive materials and cost-effective for mass production, it suffers from obvious drawbacks such as possibility of short circuit due to undesired position dislocation of electrodes and lower power density due to electron and ion transport limitations [4, 5]. In particular, the sandwich electrode configuration makes it challenging to integrate the conventional MSCs with other microelectronic devices mounted on a planar integrated circuit, which greatly limit the miniaturization of the entire microelectronic system. To address these shortcomings, the planar interdigital configuration is suggested and has become dominant in the fabrication of MSCs mainly because of its well-established advantages such as facilitating fabrication and integration. It allows the fast movement of ions in the same plane to enhance power density, and effectively prevent electrode short circuit and undesired position dislocation of electrodes [24–28]. So far, there have been various fabrication methods developed for planar MSCs, such as conventional photolithography technique [29–31], screen printing method [32], selective wetting-induced method [33], microfluidic etching assisted method [34], and laser irradiation-assisted fabrication method [35, 36]. However, most of these methods normally involve a complicated and toxic lithography process that often results in high costs, material wastage, difficulty in patterning large areas, and a certain degree of environmental pollution. Therefore, the development of convenient, low materials waste, low-cost, and environmentally friendly fabricating method for planar MSCs is significant for the further commercial application of the MSCs [5].

Inkjet printing technique is a kind of simple and high-utilization method, which can accomplish the deposition and the patterning in the same step. This will reduce material usage and process complexity [32, 37, 38]. In addition, the printing technique allows end-user to control the pattern design by propelling droplets of ink onto paper, plastic, or other substrates through simple software. Thus, it has good control in the printing precision [39]. Significantly, compared with traditional fabrication approaches, inkjet printing does not involve complicated multi-step lithography procedures, toxic chemical treatments, high temperature, and vacuum processing. It provides a simple route for fabrication of planar MSCs with high practicality and high performance. Therefore, it is a competitive alternative to conventional photolithography for the efficient, low-cost, and large-scale production of planar MSCs with high practicality for further industrial applications. Since the performance of inkjet-printable micro-electrodes of MSCs is strongly governed by the ink, preparation of liquid phase materials (active ink) that meets specific conditions (suitable viscosity and surface tension) to be printed through the micronozzles for inkjet printing is the key for the fabrication of planar MSCs [40–42].

In this field, carbon-based materials such as carbon nanotubes (CNTs) [43, 44], graphene [45–47], and graphene oxide (GO) [48–52] have received more extensive attention for the ink formulation and practical electric double-layer capacitor (EDLC) applications because of their abundant sources, large surface area, corrosion resistance, high conductivity, and suitability for mass production. However, the poor dispersibility of CNTs and graphene has greatly limited their applications. Although they can be well dispersed in some organic solvents such as dimethylformamide and *N*-methylpyrrolidone, these organic solvents are often of very low viscosity (<2 cP), which severely deteriorates the inkjetting performance [38]. In addition, these organic solvents are toxic. Furthermore, the graphene concentration in these solvents is often so low (<0.1 mg mL^−1^) that several tens of print passes are required to obtain functional devices, which can reduce efficiency of the technique. Therefore, GO with its excellent solubility in water has attracted more attentions on inkjet printing of carbon-based materials for MSCs. However, GO flakes easily aggregate and restack during solvent evaporation after printing and the following reduction processes to regain the electrical performance of pristine graphene [40]. Therefore, the actual accessible surface area of the reduced GO (rGO) electrodes is much lower compared to the theoretical surface area, which greatly degrades the performance of graphene-based MSCs. Therefore, it is essential to modify the GO ink with additives to alleviate the flake aggregation before good performance could be realized.

Herein, we formulate carbon-based hybrid ink composed of GO (98.0 vol.%) ink and commercial pen ink (2.0 vol.%), and demonstrate the fabrication of flexible and all-solid-state MSCs based on binder-free hybrid planar interdigital micro-electrodes via inkjet printing. The electrochemical properties of the as-obtained MSCs were examined by cyclic voltammetry (CV), galvanostatic charge–discharge (GCD), and electrochemical impedance spectroscopy (EIS). It was found that the as-obtained MSCs exhibit great enhancement in areal capacitance, excellent flexibility, and cycling stability. Our investigation demonstrate a promising strategy for large-scale preparation of low-cost, lightweight, and flexible/wearable energy storage devices with carbon-based hybrid ink.

## Experiment

### Preparation of Pure GO Ink

All chemicals are analytical grade and are used without further purification. GO was synthesized by oxidation of graphite with the modified Hummers method [53]. Then, the as-obtained GO aqueous solution was intensively ultra-sonicated for 60 min and filtered through a filter with 0.8 µm pore size to remove any unexfoliated GO sheets and obtained a stable GO dispersion. Finally, the GO dispersion was concentrated to the nominal concentration of 2 mg mL^−1^, and ethylene glycol (2.0 vol.%) was added to the dispersion to optimize the viscosity and surface tension.

### Preparation of Carbon-Based Hybrid Ink

The commercial pen ink (Hero, Shanghai Ink Factory in China) was firstly filtered through a filter with 0.45 µm pore size to remove any large particles. Then, the carbon-based hybrid ink was prepared by simple mixing the as-obtained pure GO ink and the commercial pen ink with the volume ratio of 49:1. The final hybrid ink was thoroughly ultra-sonicated for 5 min at room temperature.

### Inkjet Printing and Reduction

Inkjet printing was carried out with a commercial piezoelectric Dimatix material printer (DMP 2800, Dimatix-Fujifilm, Inc.) with a print head consisting of 16 inkjet nozzles designed for a 10 pL nominal drop volume. The as-obtained printing ink was injected into a cleaned ink cartridge using a syringe. Before printing, the ink cartridge was allowed to stand for several minutes to ensure that the ink was equilibrated in the cartridge, and then the patterns designed by common drawing software were printed onto the flexible PET substrates. The printing step was repeated five times to deposit sufficient active ink for electrochemical measurements. Subsequently, the printed patterns were reduced at 80 °C by HI vapor for 30 min, rinsed three times with ethanol and water in turn, and heated at 150 °C for 2 h to remove iodide ions to form the micro-electrodes of the MSCs.

### Fabrication of Flexible and All-Solid-State MSCs

Two copper wires were connected to the pad of the as-printed micro-electrode using silver paste to make a connection to the electrochemical instruments. Polyvinyl alcohol (PVA)/sulfuric acid (H_2_SO_4_) served as electrolyte was prepared by adding PVA power (6 g) into H_2_SO_4_ aqueous solution (6 g H_2_SO_4_ into 60 mL deionized water). The whole mixture was heated to 85 °C under vigorous stirring until the solution became clear. After cooling down, the gel solution was drop cast to the surface of the microelectrodes. After PVA/H_2_SO_4_ gel electrolyte being solidified, the preparation of the MSCs was completed.

**Fig. 1:**
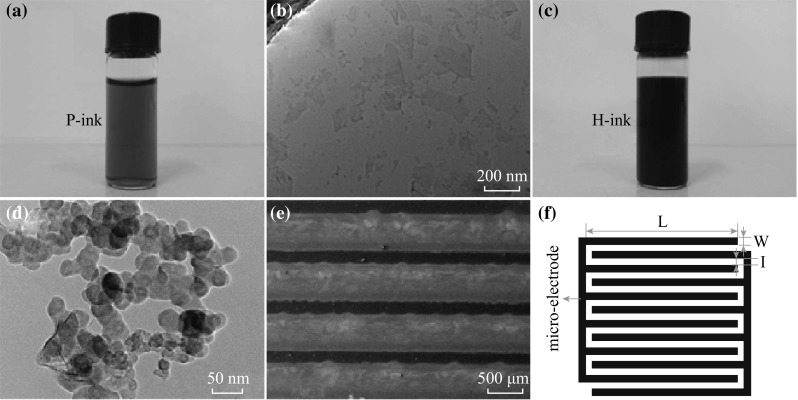
**a** GO dispersed in water at 2 mg mL^−1^ as a stable pure GO ink. **b** A typical TEM image of the GO sheets. **c** Photograph of the as-prepared carbon-based hybrid ink. **d** A typical TEM image of the graphite carbon nanoparticles dispersed in the commercial pen ink. **e** SEM image of the ink-jet printed micro-electrodes. **f** Schematic diagram of a symmetric device with 12-interdigital fingers

The areal capacitance (*C*_S_) (µF cm^−2^) was calculated from the charge–discharge curves according to the following equations to evaluate the charge storage capacity of the as-obtained MSCs.

1 $$C = Q/\Updelta E = I\Updelta t/\Updelta E,$$

2 $$C_{\text{S}} = C/S = I\Updelta t/S\Updelta E,$$

where *C* (μF) is the total capacitance, *Q* (C) is the total charge, *I* (μA) is the discharge current, *t* (s) is the discharge time, Δ*E* (V) is the potential window during the discharge process after IR drop, and *S* (cm^2^) is the total surface of the positive and the negative interdigital electrodes as shown in Fig. 1f.

### Sample Characterizations

The morphologies of all samples were characterized by scanning electron microscopy (SEM, SU-8020) and transmission electron microscopy (TEM, JEOL-2010) with an accelerating voltage of 200 kV. The Raman spectrum was taken on a LABRAM-HR confocal laser micro-Raman spectrometer using an Ar^+^ laser with the 514.5-nm line at room temperature. CV, EIS, and GCD measurements of MSCs were carried out on an electrochemical workstation (IM6ex, Zahner). Impedance spectroscopy measurements were performed at open circuit voltage with ±10 mV amplitude. Sheet resistance of the printed micro-electrode was measured by a standard four-point probe method (RST-9, Four-Probe Tech.). A Panasonic DMC-FX3 digital camera was used to capture all the photographs. All measurements were carried out at room temperature and at a relative humidity of ~60%.

## Results and Discussion

In order to obtain high-quality printed pattern and printhead reliability, restricting the size of particles dispersed in the ink is the key. The size of particles dispersed in conventional pigment ink for inkjet printing is usually in the range of 100–400 nm [54]. The initially obtained GO sheets dispersed in aqueous solution was intensively sonicated and filtered through a filter with 0.8 µm pore size to remove any unexfoliated and large GO sheets to obtain a stable GO ink. Figure 1a shows the graph of the stable pure GO ink (defined as P-ink) containing uniform and required size of GO sheets. As shown in the TEM image (Fig. 1b), after treatment, the GO sheet size is generally smaller than 400 nm, meeting the specific conditions for inkjet printing. In addition, the as-prepared P-ink can be stored for several months without any aggregation because the surface of the GO sheets contains hydrophilic functional groups [55]. The carbon-based hybrid ink was prepared by further blending pure GO ink with commercial pen ink (Fig. S1), which consists of a large number of nanoparticles about 40 nm in size as shown in Fig. 1d. Figure S2 shows the corresponding Raman spectra of the commercial pen ink, which has two characteristic bands centered at 1353 and 1582 cm^−1^ attributed to the disordered carbonaceous component and ordered graphitic component, respectively. The calculated intensity peak area ratio *I*_D_/*I*_G_ is 0.93. The Raman investigation indicates that these nanoparticles dispersed in the commercial pen ink are mainly graphite carbon nanoparticles (CNPs). Figure 1c shows homogeneous and well-dispersed black hybrid ink. This solution remains stable over a period of 3 months. The as-obtained homogeneous hybrid ink (defined as H-ink) can be easily printed on flexible PET substrate to realize large-scale fabrication of interdigital micro-electrodes for MSCs (defined as MSCs-H that was fabricated based on H-ink) as shown in Fig. S3. Figure 1e shows the good control over pattern geometry and location of MSCs-H. The well-defined pattern guarantees the absence of short circuit path between cathode and anode during the testing. Significantly, after reduction process, the as-obtained printed interdigital micro-electrodes have strong adhesion to the substrate even without a binder, which ensures the good resistance to scratch (see Supporting Information Video 1). In addition, the average sheet resistance of the printed interdigital micro-electrodes has also been measured by a standard four-point probe method, which reaches ~7.0 kΩ sq^−1^ and is higher than that (~4.3 kΩ sq^−1^) of the printed interdigital micro-electrodes based on P-ink. The increase of average resistance of the printed interdigital micro-electrodes based on H-ink is attributed to the addition of the CNPs of which the conductivity is poor. In this work, the length (*L*) and the width (*W*) of each interdigital finger were set to a constant value of 9000 and 500 μm utilizing simple software on a personal computer, respectively. The inter-space (*I*) between interdigital fingers is designed as 200 μm. However, in fact, the width of each interdigital finger and inter-space between them are only kept for an average value of 547/187 μm in the actual printing, containing a printer error relative to the set of printing parameters. If the printing parameter is set smaller, the actual printing value will contain bigger printing errors and the print quality will also deteriorate. This may cause a short circuit. Figure 1f is the schematic circuit diagram of a MSCs unit with 12-interdigital fingers. The printing step was repeated for five times to deposit sufficient active materials for electrochemical measurements. For comparison, MSCs based on P-ink and pure diluted commercial pen ink were also fabricated with the same printing parameters and denoted as MSCs-P and MSCs-CNPs, respectively.

**Fig. 2:**
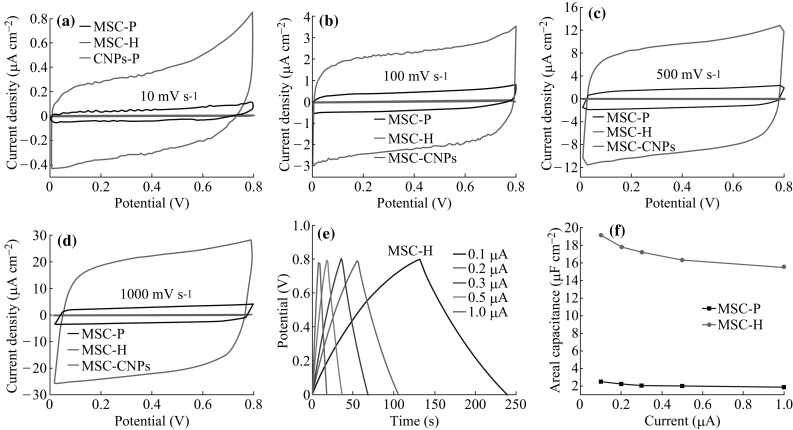
CV curves of MSCs-H, MSCs-P, and MSCs-CNPs at scan rates of: **a** 10, **b** 100, **c** 500, and **d** 1000 mV s^−1^. **e** Galvanostatic charge–discharge curves of MSC-H in the voltage range between 0 and 0.8 V at various currents. **f** Comparison of areal capacitances of MSCs-H and MSCs-P at the same current

Figure 2a–d show CV results of the as-prepared MSCs-H, MSCs-P, and MSCs-CNPs in the potential window of 0–0.8 V at various scan rates from 10 to 1000 mV s^−1^. Both MSCs-H and MSCs-P show near rectangular CV curves, typical for EDLCs. However, MSCs-H shows larger current density compared to MSCs-P at the same scan rate, indicating enhanced capacitance based on hybrid ink. In addition, compared with those of the MSCs-H and MSCs-P, the current density of MSCs-CNPs can almost be ignored at the same scan rate, which indicates that the enhanced capacitance of MSCs-H is not just derived from the added CNPs themselves. Figure 2e shows the GCD curves of MSCs-H. It can be observed that the charge and discharge time of MSCs-H increase with the trace addition of commercial pen ink at the same current, also indicating the enhanced capacitance of MSCs-H compared with that of MSCs-P (Fig. S4). The calculated areal capacitance of MSCs-H and MSCs-P from GCD curves with respect to different discharge currents is plotted in Fig. 2f. As can be seen from Fig. 2f, the areal capacitance of MSC-H was calculated to be 19.18 μF cm^−2^ at a current of 0.1 μA and 15.57 μF cm^−2^ at a current of 1.0 μA. These results achieve a high retention ratio of over 81.2%, indicating a high-rate capability. However, for MSC-P the areal capacitance is only 2.47 μF cm^−2^ at a current of 0.1 μA and 1.88 μF cm^−2^ at a current of 1.0 μA, which shows not only a lower areal capacitance but also a lower rate capability (76.1%) because of the limitation of electron/ion transfer in the close restacked graphene sheets in the absence of CNPs served as nano-spacers. The nearly 780% enhancement in areal capacitance directly confirms the remarkable advantage of using commercial pen ink to modify the pure GO ink to alleviate the flake aggregation for planar MSCs with high areal capacitance.

**Fig. 3:**
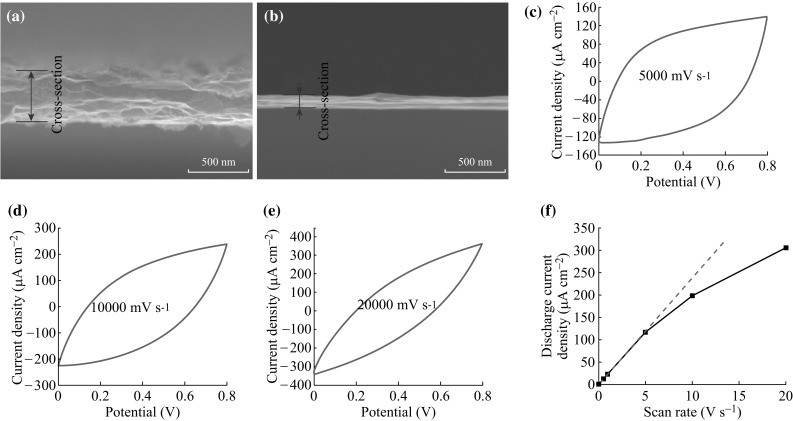
Cross-sectional SEM images of the printed micro-electrodes of MSCs: **a** MSCs-H, **b** MSCs-P. **c**–**e** Cyclic voltammetry curves of MSCs-H. **f** A linear dependence of the discharge current density on the scan rate up to 5000 mV s^−1^

We suggest that the highly enhanced areal capacitance of the MSC-H is mainly attributed to the microstructure of the electrodes. Figure 3a is a typical cross-sectional SEM image of the patterned micro-electrodes of MSCs-H. It shows the irregular porous structure of the electrodes with the appearance of CNPs between rGO sheets throughout the thickness of the films with almost no sign of stacked rGO sheets. These CNPs, served as nano-spacers, can separate neighboring GO sheets and further effectively inhibit the agglomeration during solvent evaporation after printing and restacking of graphene sheets during the reduction process, thus providing a highly accessible surface area for the microelectrodes. This loose structure is beneficial for the uptake of electrolyte to facilitate ion transport between active materials and the electrolyte, which is favorable for increasing the capacitance of EDLC [56]. On the contrary, the heavily stacked MSCs-P (Fig. 3b) could prevent the full access of electrolyte ions to the surface of rGO sheets and decrease the specific surface area of rGO, resulting in the lower areal capacitance. The rate capability and power handling of the MSCs-H was further tested by CV at high scan rates from 5000 to 20,000 mV s^−1^. Remarkably, when the scan rate further increases from 5000 to 20,000 mV s^−1^ (Fig. 3c–e), the peak current density of MSCs-H continues to increase rapidly which is the characteristic of a high instantaneous power. A linear dependence of the discharge current density on the scan rate was also recognized at least up to 5000 mV s^−1^ (Fig. 3f). As shown in Fig. 3d, e, CV curves of the MSCs-H gradually deviate from a rectangular shape as the scan rate further increases, indicating the transition to a more resistive behavior at high scan rate.

**Fig. 4:**
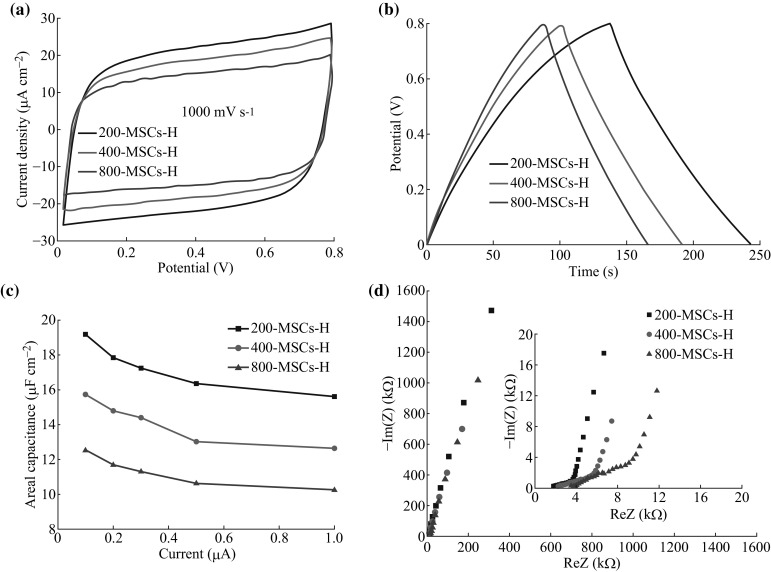
Electrochemical behaviors of 200-MSCs-H, 400-MSCs-H, and 800-MSCs-H: **a** cyclic voltammetry at a scan rate of 1000 mV s^−1^, **b** galvanostatic charge–discharge curves in the voltage range between 0 and 0.8 V at a fixed current of 0.1 μA, **c** areal capacitance plots, and **d** Nyquist plots with a frequency loop from 100 kHz to 10 mHz using a perturbation amplitude of 10 mV at the open circuit potential (*inset* shows an enlarged curve in a high-frequency region)

MSCs-H with different inter-spaces between interdigital fingers (200, 400, and 800 μm) were further fabricated to investigate the relationship between electrochemical performance and inter-space (defined as 200-MSCs-H, 400-MSCs-H, and 800-MSCs-H, respectively). Figure 4a shows CV results of the as-prepared 200-MSCs-H, 400-MSCs-H, and 800-MSCs-H in the potential window of 0–0.8 V at a constant scan rate of 1000 mV s^−1^. As shown in Fig. 4a, the current density slightly increases at the same scan rate, indicating enhanced capacitance of the device with the decrease of the inter-space between interdigital micro-electrodes. The GCD curves of MSCs-H with different inter-spaces at a constant current of 0.1 μA are shown in Fig. 4b, and the 200-MSCs-H shows obviously longer charge–discharge time compared with 400-MSCs-H and 800-MSCs-H. The calculated areal capacitance of MSCs-H with respect to different discharge currents is plotted in Fig. 4c. It shows that 200-MSCs-H has obviously larger areal capacitance compared to 400-MSCs-H and 800-MSCs-H, which can be attributed to a decrease of the inter-space between the fingers.

According to the previous reports, the smaller inter-space will reduce the ions and charge transport path that is conductive to make use of active materials completely and improve rate capability [33, 57]. Since the same electrodes (the same width and length of each interdigital finger and deposition time) were utilized for all the MSCs-H in this study, an increase in inter-space between two electrodes must result in the increase of pathway of ions transport from one electrode to the counter electrode. Thus, areal capacitance decreased as the inter-space between the interdigital finger line-width increased. The enhanced electrochemical performance of 200-MSCs-H was further confirmed by EIS measurements from 100 kHz to 10 mHz. The intercept of the Nyquist curve with the real axis at high frequencies represents the equivalent series resistance of the device with two-electrode configuration [7, 26]. From Fig. 4d, 200-MSCs-H shows a smaller intrinsic resistance than those of 400-MSCs-H and 800-MSCs-H due to the decrease of the inter-space. This is greatly important since less energy and power will be wasted to produce unwanted heat during the charge–discharge processes. Therefore, the performance of the as-presented MSCs could be further improved with a shorter inter-space between two micro-electrodes, if the viscosity and surface tension of the ink were further finely tailored to be more compatible with the printing substrate for a better inkjet printing precision.

**Fig. 5:**
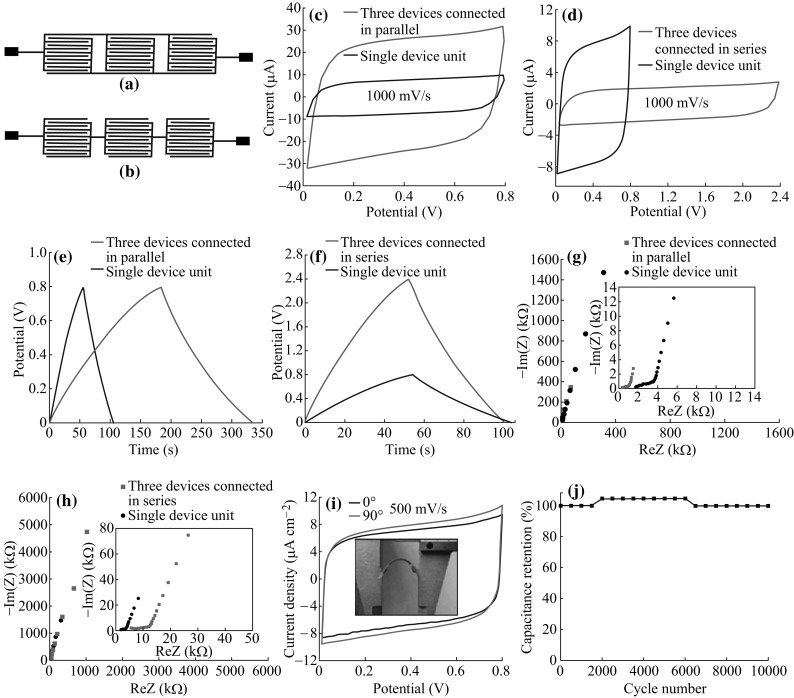
Printed circuit diagram of the flexible and all-solid-state device connected **a** in parallel and **b** in series. Cyclic voltammetry curves for a single device and three devices connected in parallel (**c**) and in series (**d**) at a high scan rate of 1000 mV s^−1^. Galvanostatic charge–discharge curves for a single device and three devices connected in parallel (**e**) and in series (**f**) at the same constant current of 0.2 μA. Nyquist plots of three devices connected in parallel (**g**) and in series (**h**) with a frequency loop from 100 kHz to 10 mHz using perturbation amplitude of 10 mV at the open circuit potential (*insets* in **g** and **h** show enlarged views at high-frequency regions). **i** Cyclic voltammetry curves of the device under normal and bent conditions at a scan rate of 500 mV s^−1^, and the *inset* shows a photograph of the bent device, and **j** long-term cycling stability of the device measured at a current of 1 μA cm^−2^

Considering practical use of MSCs, it is needed to connect SCs in series and/or in parallel to increase operating voltage and capacity in some situations. The performance of integrated MSCs pack with three 200-MSCs-H connected in parallel and series were tested. Figure 5a, b is the photographs of printed three devices connected in parallel and series. Figure 5c shows the CV curve of single device and the pack (three devices connected in parallel) at 1000 mV s^−1^ with a potential window of 0–0.8 V, where the output current of the pack increases by a factor of ~3 compared with a single device. In addition, as shown in GCD curves of a single device and the pack at the same constant current of 0.2 μA (see Fig. 5e), the runtime of the pack also increases by a factor of ~3 compared with a single device. The calculated capacitance of the electrochemical capacitor pack and a single device from GCD curves are 12.36 and 37.77 μF, respectively, revealing that the device roughly obeys the basic rule of parallel connections. The intercept of the Nyquist curve with the real axis at high frequencies represents the equivalent series resistance of the device [8, 10]. As shown in Fig. 5g, the inner resistance of three parallel-connected devices is only about a third of single device unit. Therefore, putting the devices together in parallel can effectively enhance the output current and the capacitance, while the operating voltage still remains the same. Figure 5d is the CV curves of single device unit and three devices units connected in serial at a high scan rate of 1000 mV s^−1^. One can see adding electrochemical capacitors in a serial string can effectively increase the voltage when portable equipments need higher voltages. However, due to the inner resistance was superimposed after MSCs units connected in series (Fig. 5h), the current of three devices units connected in series is reduced compared with the single device unit. The calculated capacitance of the pack is 4.04 μF, which is about one third of that of the single device (12.36 μF). These results show a good consistency of the electrochemical performance of the on-chip and all-solid-state MSCs based on all-carbon-based hybrid ink. The as-obtained planar on-chip MSCs also possess excellent mechanical flexibility, which can be bent outwards to 90° at least (radius of curvature is 4.6 mm) and still remain the CV curves nearly unchanged compared with that of the normal configuration at a high scan rate of 500 mV s^−1^ (Fig. 5i). Furthermore, the CV curves after repeatedly bending for 500 times also remain nearly unchanged at a high scan rate of 500 mV s^−1^ as shown in Fig. S5. All the results demonstrate its potential application in flexible energy storage. As a potential energy storage device, the long-term stability of MSCs should also be examined. The cycling performance of the device was further tested by GCD at a current of 1 μA over 10,000 cycles. As shown in Fig. 5j, after the initial 1500 cycles, a small increase of capacitance is observed for the subsequent cycles. This phenomenon is due to the incomplete exposure of active sites of the printed micro-electrodes to the gel electrolyte [58–60]. The capacitance of the device decreases to the initial capacitance, indicating the full infiltration of the gel electrolyte to the active sites. However, there is almost no attenuation in the capacitance of the device as the number of cycle increases to 10,000 and the capacitance still retains about 100% after 10,000 cycles with respect to the first cycle, which demonstrates its excellent electrochemical stability.

## Conclusions

In summary, it is shown that carbon-based hybrid ink composed of GO ink and commercial pen ink can be formulated successfully. A commercially scalable inkjet printing technique can be used to prepare flexible and all-solid-state MSCs without using traditional lithography or screen printing method. The MSCs formed with the binder-free hybrid planar interdigital micro-electrodes exhibit nearly 780% enhancement in areal capacitance compared to that of printed devices using pure GO ink. It also has excellent flexibility and cycling stability. Moreover, these all-solid-state devices can be optionally connected in series or in parallel to meet the voltage and capacity requirements for a given application. The key result of this work is the applicability of the inkjet printing technique used carbon-based hybrid ink from cheap, abundant, commercially available materials to allow an industrially scalable route to achieve printable energy storage devices with high performance.

## Electronic supplementary material

Below is the link to the electronic supplementary material.

Supplementary material 1 (PDF 391 kb)

Supplementary material 2 (WMV 1076 kb)
