# Supplementary material for: Carbon-Based Flexible and All-Solid-State Micro-supercapacitors Fabricated by Inkjet Printing with Enhanced Performance
Source: Nanomicro Lett. 2016 Dec 8;9(2):19. doi: 10.1007/s40820-016-0119-z (PMC6223797; doi:10.1007/s40820-016-0119-z)
Supplement: Supplementary file 1 — Supplementary material 1 (PDF 391 kb) [file 40820_2016_119_MOESM1_ESM.pdf]

Supporting information for

Carbon-Based Flexible and All-Solid-State Micro-Supercapacitors Fabricated by Inkjet Printing with Enhanced Performance

Zhibin Pei[1,2] , Haibo Hu[1,*], Guojin Liang[1], Changhui Ye[1,*]

[1]Anhui Key Laboratory of Nanomaterials and Technology, and Key Laboratory of Materials Physics, Institute of Solid State Physics, Chinese Academy of Sciences, Hefei 230031, People's Republic of China

[2]University of Science and Technology of China, Hefei 230026, People's Republic of China

[3]Hubei Collaborative Innovation Center for Advanced Organic Chemical Materials, Ministry-of-Education Key Laboratory for the Green Preparation and Application of Functional Materials, Faculty of Materials Science and Engineering, Hubei University, Wuhan 430062, People's Republic of China

*Corresponding authors. E-mail: chye@issp.ac.cn, haibohu@issp.ac.cn

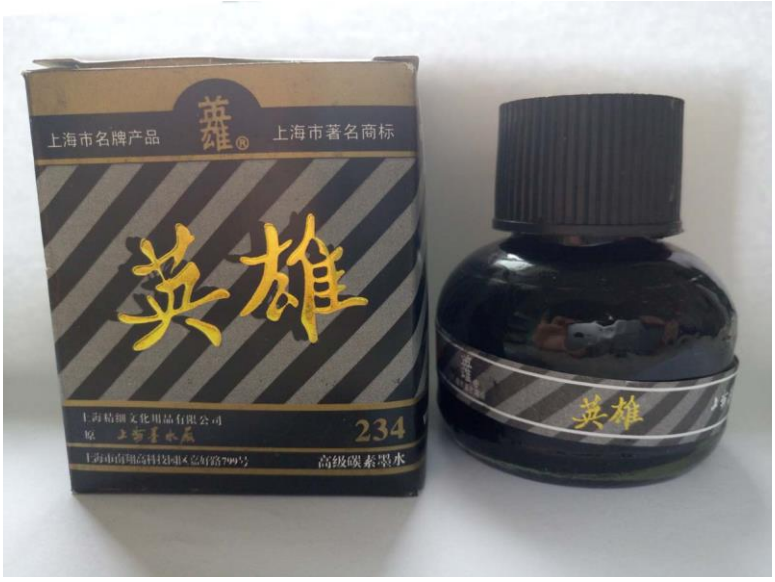

Figure S1 A photograph of the commercial pen ink

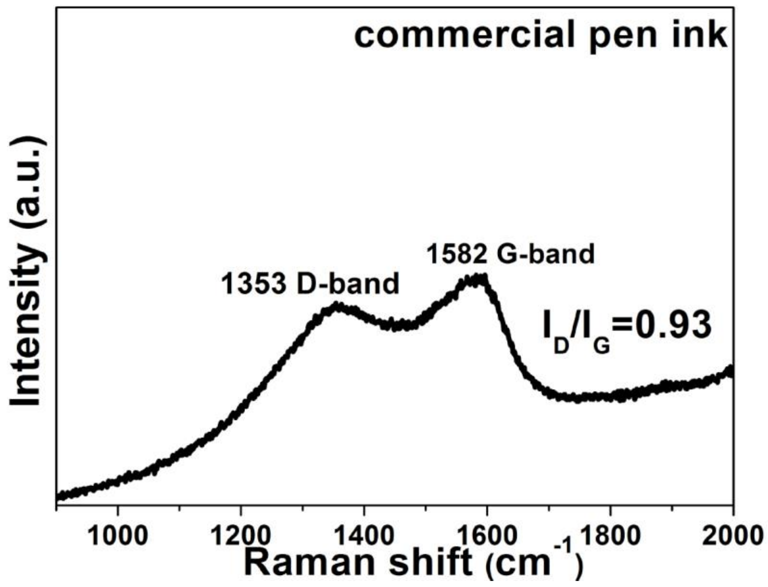

Fig. S2 Raman spectra of the commercial pen ink

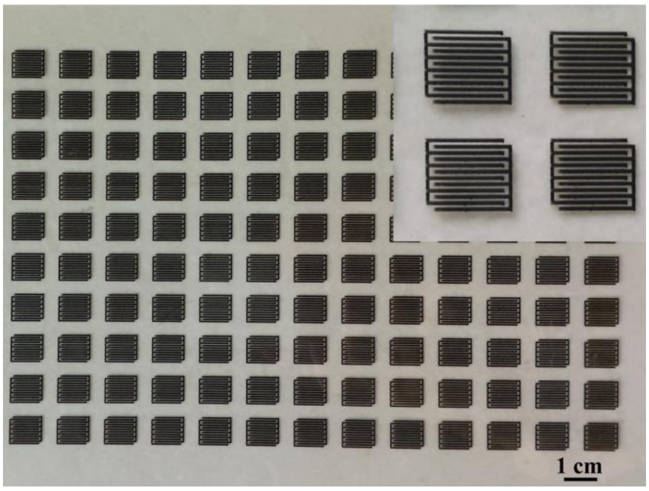

Fig. S3 A photograph of the directly large-scale ink-jet printed MSCs-H with the carbon-based hybrid ink on a flexible PET film (inset shows an enlarged photograph)

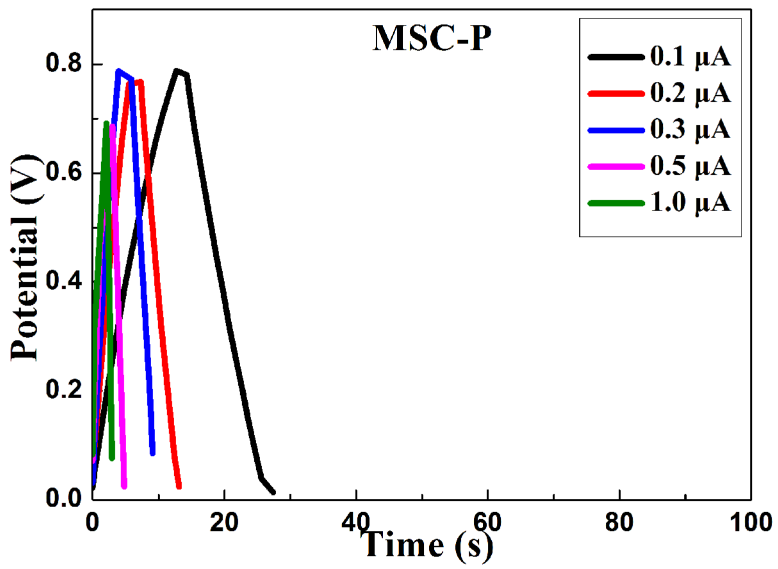

Fig. S4 Galvanostatic charge–discharge curves of MSC-P in the voltage range between 0 and 0.8 V at various current

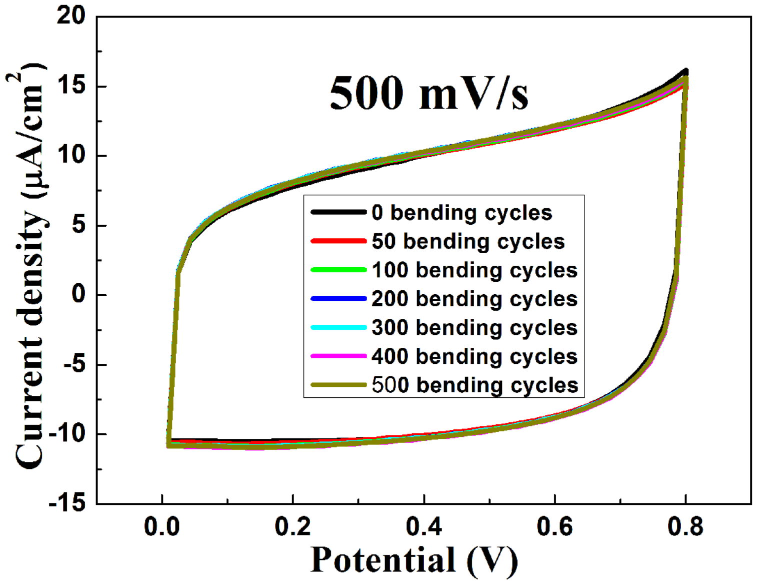

Fig. S5 Cyclic voltammetry curves of the device after repeatedly bending for 500 times at a scan rate of 500 mV s^{-1}
